# Clinical predictors of successful vaginal myomectomy for prolapsed pedunculated uterine leiomyoma

**DOI:** 10.4274/jtgga.2017.0135

**Published:** 2018-08-06

**Authors:** Serdar Aydın, Hale Göksever Çelik, Mustafa Maraşlı, Rabia Zehra Bakar

**Affiliations:** 1 Department of Obstetrics and Gynecology, Bezmialem Vakıf University School of Medicine, İstanbul, Turkey; 2 Department of Obstetrics and Gynecology, Saglik Bilimleri University, İstanbul Kanuni Sultan Süleyman Training and Research Hospital, İstanbul, Turkey; 3 Department of Obstetrics and Gynecology, Erbaa State Hospital, Tokat, Turkey

**Keywords:** Leiomyoma, prolapsed pedunculated submucosal leiomyoma, vaginal myomectomy, hysterectomy

## Abstract

**Objective:**

Uterine leiomyomas are the most common pelvic tumor in women. The calculated prevalence of prolapsed pedunculated leiomyoma was 2.5% in patients who underwent surgery. Although vaginal removal is safe and effective, hysterectomy demand is questionable. We aimed to analyze the association between patient characteristics, clinical features of prolapsed pedunculated submucosal leiomyoma, and the probability of successful vaginal myomectomy.

**Material and Methods:**

This study was conducted in 35 women who presented with prolapsed pedunculated uterine leiomyoma. Patients were grouped according to the treatment procedure, either vaginal myomectomy or hysterectomy.

**Results:**

Hysterectomy was performed in 14 patients and vaginal myomectomy was performed in 21 women. The mean ages and menopausal status were similar. Parity was higher in the hysterectomy group (p=0.02). The preoperative hematocrit value of patients undergoing vaginal myomectomy was significantly lower (p=0.04). There was no significant difference between the groups regarding the largest leiomyoma diameter. However, the median calculated leiomyoma volume was lower in the vaginal myomectomy group (p=0.04). None of the variables were independently associated with successful vaginal myomectomy on multivariable logistic regression analysis.

**Conclusion:**

The feasibility and choice of vaginal myomectomy is associated with low parity, absence of coexisting leiomyoma, high volume of leiomyoma estimated via ultrasound measurement, and severe anemia.

## Introduction

Uterine leiomyomas are the most common pelvic tumor in women arising from the smooth muscle of the myometrium (1). Leiomyomas are classified according to their location within the myometrium. Prolapsed pedunculated submucous myoma is classified as type 0 submucous leiomyoma (2). In general, the prevalence of uterine leiomyoma is approximately 25% of reproductive-aged women and submucosal leiomyoma account for approximately 15-20% of these (3). The real prevalence of prolapsed pedunculated submucosal myoma is unknown but the calculated prevalence was 2.5% in patients who underwent surgery for leiomyoma (4). They typically present with vaginal bleeding, pelvic pain that is generally cramping in nature during the expulsion of the myoma from the cervix, and dysmenorrhea and bloody discharge. Surgery is recommended for symptomatic patients. Although vaginal removal is safe and effective for uterine prolapsed pedunculated leiomyoma, hysterectomy demand is questionable (4,5,6).

Few series in the English literature about the management of uterine prolapsed pedunculated submucous myomas have been reported (4,5,6,7,8,9). However, this report mainly addresses the early results, complications, and feasibility of vaginal removal. There is an evidence gap regarding how patient characteristics and leiomyoma features influence the choice of operative procedures. Better understanding of factors that influence the probability of undergoing hysterectomy would help deliver patient-centric uterine leiomyoma care. The aim of this study was to analyze the association of patient characteristics and the probability of hysterectomy and the results of the two procedures in patients with prolapsed pedunculated submucosal myoma.

## Material and Methods

This retrospective cross-sectional study was based on the analysis of medical records of 35 women who presented with prolapsed pedunculated uterine leiomyoma. We reviewed the hospital records of all patients admitted to departments of gynecology of two centers with a clinical diagnosis of prolapsed pedunculated submucous myoma between November 2011 and March 2016. The local ethics committee approved the study.

Demographic, clinical and pathologic data, including age, gravidity, parity, history of uterine surgery and cesarean section, smoking status, menopausal status, medical history, complications, and preoperative and postoperative hematocrit were all retrieved from the medical records. A history of medical conditions, including hypertension, diabetes mellitus, hypercholesterolemia and thyroid disease were recorded if hospital records confirmed the diagnosis. Patients with amenorrhea for more than one year since the last menstrual period were considered to be menopausal. 

Only myomas with a pedicle base originating at or above the level of the internal cervical os were included. There was a total of 35 patients during the study period. Patients were grouped according to the performed procedure; either vaginal myomectomy or hysterectomy. Vaginal myomectomy was performed with general anesthesia under sterile conditions. The myoma was grasped using tenaculum forceps under direct vision and twisted around its pedicle. Too much downward traction on the myoma was avoided so as not to cause inversion of the uterus. In some cases, when feasible, the pedicle was clamped and ligated as high as possible. Myomectomy was successfully accomplished without dilatation of the cervix. Total abdominal hysterectomy with or without concomitant bilateral salpingo-oophorectomy were performed using a standard clamp technique as performed in benign gynecologic indications. All patients in whom hysterectomy was performed were decided at the beginning of the patients’ examinations. If laparoscopic hysterectomies were performed, vaginal removal of the leiomyoma was performed first to place the uterine manipulator.

### Statistical analysis

Data are expressed as mean ± standard deviation or number and percentage, median and range, as appropriate. Statistical analysis was performed after normality testing (histogram analysis and/or Kolmogorov–Smirnov) using IBM SPSS version 22. Student’s t-test was used for comparisons of normally distributed variables, and the Mann-Whitney U test was used for categorical variables. Chi-square and Fisher’s exact tests were used to compare the proportion of categorical variables. Multivariable logistic regression models were developed to predict the probability of successful vaginal removal of a prolapsed pedunculated leiomyoma using variables identified during univariate analysis. Odds ratios with 95% confidence intervals were also calculated. The Statistical Package for Social Sciences (SPSS) for Windows version 22.0 (SPSS Inc., Chicago, IL, USA) was used for the analysis and two-sided p value of <0.05 was considered as significant.

## Results

Vaginal myomectomy was successful in 21 women. Hysterectomy for prolapsed pedunculated submucosal leiomyoma was performed in 14 patients. The mean age of the study population was 46.1±8 years (range, 29-62 years). Parity was 2.7±1.7 (range, 0-7). Two women were nulliparous (5.7%) and one was nulligravida (2.8%). Only 4 women were in the postmenopausal period (11.4%). The patient characteristics, laboratory and imaging features of patients undergoing vaginal myomectomy or hysterectomy for prolapsed pedunculated vaginal leiomyoma are presented in Table 1. The mean ages were 44.8±9.2 and 47.9±5.4 years in the vaginal myomectomy and hysterectomy groups, respectively. The mean parity of the hysterectomy group was statistically higher than the uterus-sparing group (3.6±1.6 vs 2.1±1.6, p=0.02). All of the hysterectomy group patients were parous and 2 (9.5%) women in the vaginal myomectomy group were nulliparous (p=0.2). One woman (7.1%) in the hysterectomy group and 3 women (14.3%) in the vaginal myomectomy group were postmenopausal (p=0.5). Also, comorbidity profiles of the patients in both groups were similar.

On admission, hematocrit value of the patients undergoing vaginal myomectomy (29.9±5.2) was significantly lower than those undergoing hysterectomy (33.4±3.8, p=0.04). Blood transfusion prior to surgery because of preoperative severe anemia was performed to 11 patients (52.6%) in the vaginal myomectomy group and 5 patients (35.7%) in the hysterectomy group (p=0.3). 

All patients who underwent vaginal myomectomy had no more than one prolapsed pedunculated leiomyoma. The total leiomyoma number in the hysterectomy group ranged from 1 to 5 with median number of 1 (p=0.03). Median of the largest diameter of prolapsed pedunculated submucosal leiomyoma was 5 cm (range 2-10 cm) in the vaginal myomectomy group and 5.5 cm (range, 3-13 cm) in the hysterectomy group (p=0.2). The median leiomyoma volume was 60 cm^3^ (range, 24-660 cm^3^) in the vaginal myomectomy group and 112 cm^3^ (range, 40-910 cm^3^) in the hysterectomy group (p=0.04).

Multivariable logistic regression models were developed to predict hysterectomy using variables parity, preoperative hematocrit value, leiomyoma number, and estimated leiomyoma volume, which were identified during the univariate analysis (Table 2). None of the variables were independently associated with successful vaginal myomectomy or those undergoing hysterectomy. 

The mean duration of hospitalization in women who underwent hysterectomy was 6.7±3 days, whereas the duration was 3.5±3 days in the vaginal myomectomy group (p=0.005). The mean postoperative hospital stay durations were 1.7±1.4 days in vaginal myomectomy group and 3.1±1.6 days in hysterectomy group. However, the overall hospitalization (4 days, p=0.7) and the postoperative hospitalization (2±0.8, p=0.7) for the small subgroup of laparoscopic myomectomy were similar to vaginal myomectomy. Postoperative hematocrit values were similar. We did not encounter any major complications except a case of febrile morbidity seen after vaginal removal because of abrupt bleeding controlled with pedicle ligation.

## Discussion

The preference of vaginal removal or hysterectomy in patients with prolapsed pedunculated submucosal leiomyoma depends on many factors. There is an association between patient characteristics and the choice of management. Younger age and parity, lower preoperative hematocrit levels, smaller leiomyoma diameter and volume were observed in the vaginal myomectomy group, which resulted in shorter hospitalization time and postoperative hospitalization duration. 

The least invasive management option for women with symptomatic prolapsed pedunculated submucosal leiomyoma, in other words myoma in status nascendi, was evaluated in a few retrospective studies. These studies claimed that vaginal removal of prolapsed pedunculated leiomyoma was a safe and simple procedure with shorter hospitalization and minimal morbidity (4,5,6). The most probable potential complications of vaginal removal of prolapsed pedunculated leiomyoma are excessive hemorrhage from the pedicle, infection, and uterine inversion due to excessive traction. However, they did not report any complications in these series. Also, in our series, we encountered no major complications except a case of febrile morbidity controlled with antipyretic treatment and a minimal surgical procedure, as mentioned before. 

Vaginal removal of prolapsed pedunculated leiomyoma appears to be feasible in most cases (5,6). Until now, there has been no analysis of factors to predict successful vaginal removal. Although without evidence, the widely accepted features of prolapsed pedunculated leiomyoma that cannot be removed through vaginal myomectomy are leiomyoma with broad-based pedicle, non-visualized pedicle, leiomyoma larger than 4 cm, and cervical leiomyoma rather than submucosal. In a retrospective series of 46 women with prolapsed pedunculated leiomyoma, only two cases failed due to the difficulty of reaching the pedicle of the leiomyoma (6). Caglar et al. (10) reviewed 70 patients retrospectively. They concluded that leiomyoma diameter over 5 cm could not be successfully managed vaginally. They could not perform logistic regression or other statistical analysis to detect predictors, and they did not explain the rationale of choosing the diameter of 5 cm. In another retrospective series, vaginal myomectomy, abdominal myomectomy or hysterectomy were compared in the management of prolapsed pedunculated leiomyoma, but the rate of conversion from a vaginal removal to abdominal procedure was not reported (5). Even very large leiomyomas, up to 10 cm, were successfully removed vaginally and completed without any complications. In our vaginal myomectomy group, 62% of women had a leiomyoma diameter larger than 5 cm. Ultrasound estimated leiomyoma volume may help to predict vaginal myomectomy on univariate analysis, but on logistic regression it loses its importance as an independent determinant.

Surgery has been the mainstay of leiomyoma treatment. Leiomyoma, irrespective of location, was reported as the most common indication for hysterectomy according to the literature (11,12,13). Hysterectomy has the advantages of the elimination of symptoms and no risk of recurrence. Also, significant and sustained improvements are seen for symptoms, psychological function and quality of life after hysterectomy (14). Uncertainty remains regarding how different patient characteristics are associated with uterine-sparing procedures and hysterectomy (15). The choice of procedures depends on the age of the woman, reproductive potential, accompanied diseases, and anemia. The preference for hysterectomy has decreased with infertility, higher income and education, and decreasing age (16). Furthermore, concomitant menstrual disorders, uterovaginal prolapse, and previous myomectomy history increase the need for hysterectomy (17,18,19). We found that low parity, preoperative anemia, and absence of coexisting leiomyomas were associated with preferences for uterine-sparing surgery. Hysterectomy is usually required in women with multiple leiomyomas. Dicker et al. (5) presented a series of 142 patients managed with different methods, either vaginally or abdominally over 10 years. A total of 46 women had a vaginal myomectomy, 12 had abdominal hysterectomy, usually following vaginal removal of the prolapsed pedunculated leiomyoma, and 18 underwent vaginal hysterectomy. Riley (8) presented 41 patients, 13 were treated by abdominal hysterectomy. The authors reported that hysterectomy was related to slightly higher postoperative morbidity, but comparable to that of hysterectomy for other indications. As expected, the duration of hospitalization after vaginal myomectomy is shorter than with an abdominal operation (5,10). In our series, hospital stay was longer with abdominal hysterectomy, but was comparable with vaginal removal in the small series of laparoscopic hysterectomy. 

In a broad sense, the disadvantage of myomectomy is the risk of recurrence or formation of new leiomyomas. The risk of a second operation after myomectomy ranges from 11% to 26% (20,21). The detection of new leiomyomas through imaging is about 50% of women within 5 years after abdominal myomectomy (22). Although the risk of leiomyoma recurrence may be attributed to a prolapsed pedunculated leiomyoma, myoma-related symptoms appear to occur infrequently. A retrospective series of 46 women reported that 20% of cases continued to be symptomatic, 9% cases required a repeat vaginal myomectomy, and 6% had a hysterectomy at 5.5 year follow-up (4). Hysterectomy in these cases may have been performed due to indications other than the original prolapsed leiomyoma. In another series of 46 women, hysterectomy was required in an additional 14% of patients in whom vaginal removal of prolapsed pedunculated leiomyoma was successful. 

The present study has several limitations; first, the retrospective design of the study and lack of follow-up of the patients, and secondly, the relatively small sample size of the study population. The strengths of our study include new data regarding the prediction of success or choice of vaginal removal for prolapsed pedunculated leiomyomas that presented with prolapse from the cervical canal with more preoperative factors than previous studies, in the setting of laparoscopic hysterectomy.

In conclusion, both vaginal removal and hysterectomy are safe procedures for prolapsed pedunculated leiomyoma. The feasibility and choice of vaginal removal or hysterectomy depend on many factors. Lower parity, absence of coexisting leiomyoma, lower volume of presenting leiomyoma estimated via ultrasound measurement, and more severe anemia, which may be a sign that less stable hemodynamics were associated with preference of vaginal removal. However, neither of these factors predicted the operation choice or obligation independently.

## Figures and Tables

**Table 1:**
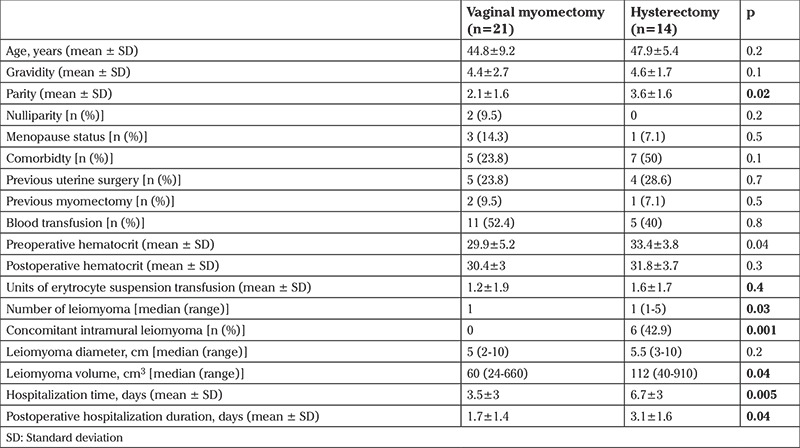
Demographic, baseline characteristics and clinical features of patients in vaginal myomectomy and hysterectomy

**Table 2:**
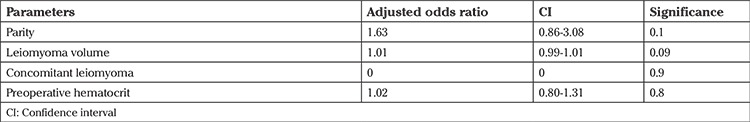
Multivariable logistic regression analysis of factors for the prediction of undergoing hysterectomy
